# Comparison of US Hospital Cash Prices and Commercial Negotiated Prices for 70 Services

**DOI:** 10.1001/jamanetworkopen.2021.40526

**Published:** 2021-12-21

**Authors:** John (Xuefeng) Jiang, Martin A. Makary, Ge Bai

**Affiliations:** 1 Accounting and Information Systems, Broad College of Business, Michigan State University, East Lansing; 2 Department of Surgery, Johns Hopkins School of Medicine, Baltimore, Maryland; 3 Johns Hopkins Carey Business School, Baltimore, Maryland; 4 Health Policy and Management, Johns Hopkins Bloomberg School of Public Health, Baltimore, Maryland

## Abstract

This economic evaluation uses national pricing information to compare US hospital cash prices and commercial negotiated prices for 70 high-volume common services specified by the Centers for Medicare and Medicaid Services.

## Introduction

On January 1, 2021, the Centers for Medicare and Medicaid Services (CMS) implemented the Hospital Price Transparency Final Rule to promote price competition and improve hospital care affordability.^1^ Hospitals in the US are required to disclose, among other items, the cash prices and the payer-specific negotiated prices for 70 CMS-specified, high-volume common services; however, the compliance rate has remained low.^1,2,3^

Cash prices can affect the cost exposure of 26 million uninsured individuals and concern nearly one-third of US workers enrolled in high-deductible health plans, who are often responsible to pay for medical bills without a third-party contribution and thus are interested in having access to low cash prices.^4,5,6^ In contrast with the commercial price negotiated bilaterally between hospitals and insurers providing insurance plans, the cash price is determined unilaterally by the hospital and might be expected to be higher than negotiated prices. The relationship between these 2 prices, however, remains largely unexplored because of a lack of available data. Using decision analytical modeling and recently disclosed pricing information, we conducted a national analysis to compare US hospital cash prices with commercial negotiated prices for 70 CMS-specified services.

## Methods

This economic evaluation was exempt from institutional review board approval because it did not meet criteria for human participant research, in accordance with the Common Rule. This study followed the Consolidated Health Economic Evaluation Reporting Standards (CHEERS) reporting guideline.

The 70 CMS-specified hospital services represent 74 unique *Current Procedural Terminology* (*CPT*)/diagnosis related group codes (4 services were represented by 2 codes). Cash prices and payer-specific negotiated prices for the 70 services were obtained from Turquoise Health, a data service company that specializes in collecting pricing information from hospitals.

For each service, hospitals that disclosed both the cash price and the commercial negotiated price were included in the sample. The following were calculated for these hospitals: (1) the median and interquartile cash prices and (2) the proportion of hospitals for which the cash price was lower than its median commercial negotiated price, lower than all commercial negotiated prices, or the same as the lowest commercial negotiated price. The Spearman test was used to assess correlations. Statistical significance was set at *P* < .001 (2-sided). Statistical analyses were conducted with SAS, version 9.4 (SAS Institute Inc).

## Results

As of July 1, 2021, 5359 hospitals had been reviewed by Turquoise Health. Of these, a mean (SD) of 922 (488) hospitals from 49 states had disclosed both their cash price and commercial negotiated price across the 70 CMS-specified services (Table). The number of disclosing hospitals and the service’s median cash price were negatively correlated (Spearman correlation coefficient, 0.39; *P* < .001). Cash prices varied substantially across hospitals for most services, as evidenced by large IQRs.

**Table.** Comparison of Hospital Cash Prices and Commercial Negotiated Prices for 70 Centers for Medicare and Medicaid Services–Specified Services

Service^a^	*CPT*/DRG code	No. of hospitals (No. of states)^b^	Cash price, median (IQR), $^c^	No. (%) of hospitals		
				Cash price less than median commercial price^d^	Cash price less than all commercial prices^e^	Cash price equal to minimum commercial price^f^
Evaluation and management services						
Psychotherapy, 30 min	90832	581 (50)	141 (92-182)	322 (55.4)	96 (16.5)	18 (3.1)
Psychotherapy, 45 min	90834	592 (50)	171 (111-263)	325 (54.9)	109 (18.4)	18 (3.0)
Psychotherapy, 60 min	90837	547 (50)	199 (140-303)	283 (51.7)	87 (15.9)	16 (2.9)
Family psychotherapy, not including patient, 50 min	90846	447 (48)	183 (117-296)	237 (53.0)	66 (14.8)	19 (4.3)
Family psychotherapy, including patient, 50 min	90847	499 (50)	203 (129-310)	257 (51.5)	66 (13.2)	17 (3.4)
Group psychotherapy	90853	526 (50)	122 (76-208)	302 (57.4)	78 (14.8)	20 (3.8)
New patient office or other outpatient visit, typically 30 min	99203	897 (50)	166 (108-254)	451 (50.3)	127 (14.2)	28 (3.1)
New patient office or other outpatient visit, typically 45 min	99204	893 (50)	203 (123-326)	474 (53.1)	149 (16.7)	31 (3.5)
New patient office or other outpatient visit, typically 60 min	99205	815 (50)	246 (142-411)	439 (53.9)	133 (16.3)	27 (3.3)
Patient office consultation, typically 40 min	99243	348 (47)	158 (116-264)	195 (56.0)	69 (19.8)	8 (2.3)
Patient office consultation, typically 60 min	99244	334 (48)	205 (140-365)	183 (54.8)	61 (18.3)	9 (2.7)
Initial new patient preventive medicine evaluation for patient aged 18-39 y	99385	365 (48)	152 (95-228)	183 (50.1)	52 (14.2)	8 (2.2)
Initial new patient preventive medicine evaluation for patient aged 40-64 y	99386	347 (48)	166 (106-278)	178 (51.3)	53 (15.3)	9 (2.6)
Laboratory and pathology services						
Basic metabolic panel	80048	1498 (51)	78 (36-137)	625 (41.7)	121 (8.1)	45 (3.0)
Blood test, comprehensive group of blood chemicals	80053	1494 (51)	108 (47-187)	598 (40.0)	115 (7.7)	45 (3.0)
Obstetric blood test panel	80055	579 (47)	187 (121-295)	276 (47.7)	59 (10.2)	16 (2.8)
Blood test, lipids (cholesterol and triglycerides)	80061	1521 (51)	84 (43-142)	616 (40.5)	114 (7.5)	39 (2.6)
Kidney function panel test	80069	1459 (51)	80 (37-138)	575 (39.4)	113 (7.7)	45 (3.1)
Liver function blood test panel	80076	1534 (51)	85 (39.6-145)	589 (38.4)	111 (7.2)	45 (2.9)
Manual urinalysis test with examination using a microscope	81000	596 (51)	20 (10-38)	246 (41.3)	56 (9.4)	21 (3.5)
Manual urinalysis test with examination using a microscope	81001	1444 (51)	36 (16-58)	593 (41.1)	103 (7.1)	42 (2.9)
Automated urinalysis test	81002	1057 (51)	17 (9-30)	466 (44.1)	94 (8.9)	32 (3.0)
Automated urinalysis test	81003	1436 (51)	25 (10-40)	614 (42.8)	115 (8.0)	48 (3.3)
PSA	84153	1494 (51)	82 (51-127)	640 (42.8)	137 (9.2)	42 (2.8)
PSA	84154	1307 (51)	66 (46-110)	628 (48.0)	160 (12.2)	35 (2.7)
Blood test, TSH	84443	1563 (51)	87 (48-137)	653 (41.8)	124 (7.9)	47 (3.0)
Complete blood cell count, with differential white blood cells, automated	85025	1494 (51)	58 (28-92)	636 (42.6)	112 (7.5)	37 (2.5)
Complete blood cell count, automated	85027	1456 (51)	46 (23-78)	619 (42.5)	116 (8.0)	43 (3.0)
Blood test, clotting time	85610	1553 (51)	34 (18-55)	632 (40.7)	117 (7.5)	50 (3.2)
Coagulation assessment blood test	85730	1464 (50)	47 (28-81)	623 (42.6)	108 (7.4)	48 (3.3)
Radiology services						
CT scan, head or brain, without contrast	70450	1604 (51)	1037 (595-1525)	645 (40.2)	138 (8.6)	46 (2.9)
MRI scan of brain before and after contrast	70553	1565 (51)	2306 (1394-3517)	631 (40.3)	146 (9.3)	47 (3.0)
Radiography, lower back, minimum 4 views	72110	1570 (51)	329 (206-509)	696 (44.3)	152 (9.7)	31 (2.0)
MRI scan of lower spinal canal	72148	1584 (51)	1625 (968-2431)	662 (41.8)	148 (9.3)	42 (2.7)
CT scan, pelvis, with contrast	72193	1492 (51)	1308 (803-1887)	615 (41.2)	143 (9.6)	47 (3.2)
MRI scan of leg joint	73721	1530 (51)	1534 (967-2298)	655 (42.8)	153 (10.0)	47 (3.1)
CT scan of abdomen and pelvis with contrast	74177	1598 (51)	2268 (1278-3534)	624 (39.0)	127 (7.9)	40 (2.5)
Ultrasonography of abdomen	76700	1584 (51)	560 (308-816)	667 (42.1)	141 (8.9)	39 (2.5)
Abdominal ultrasonography of pregnant uterus single or first fetus	76805	1305 (50)	464 (286-686)	627 (48.0)	129 (9.9)	47 (3.6)
Ultrasonography of pelvis through vagina	76830	1565 (51)	406 (246-619)	716 (45.8)	162 (10.4)	40 (2.6)
Mammography of 1 breast	77065	1241 (50)	225 (157-323)	659 (53.1)	179 (14.4)	43 (3.5)
Mammography of both breasts	77066	1247 (50)	277 (196-404)	643 (51.6)	162 (13.0)	38 (3.0)
Mammography, screening, bilateral	77067	1298 (50)	233 (158-333)	681 (52.5)	197 (15.2)	37 (2.9)
Medicine and surgery services						
Cardiac valve and other procedures with cardiac catheterization with comorbidities	216	309 (44)	127 976 (78 402-212 558)	175 (56.6)	56 (18.1)	11 (3.6)
Spinal fusion except cervical without complications (MCC)	460	491 (47)	51 366 (32 329-91 380)	242 (49.3)	91 (18.5)	13 (2.6)
Major joint replacement or reattachment of lower extremity without complications	470	710 (49)	29 097 (17 735-45 214)	375 (52.8)	131 (18.5)	13 (1.8)
Cervical spinal fusion without complications	473	446 (46)	34 044 (20 330-62 430)	205 (46.0)	83 (18.6)	15 (3.4)
Uterine and adnexa procedures for nonmalignancy without complications (MCC)	743	571 (49)	18 429 (10 561-32 103)	277 (48.5)	86 (15.1)	15 (2.6)
Removal of ≥1 breast growth, open procedure	19120	671 (49)	3677 (2311-6112)	339 (50.5)	108 (16.1)	6 (0.9)
Shaving of shoulder bone using an endoscope	29826	361 (47)	4584 (1999-15 310)	150 (41.6)	39 (10.8)	4 (1.1)
Removal of 1 knee cartilage using an endoscope	29881	603 (49)	4912 (3142-10 143)	299 (49.6)	95 (15.8)	6 (1.0)
Removal of tonsils and adenoid glands of patient younger than 12 y	42820	415 (47)	4573 (2329-6718)	212 (51.1)	81 (19.5)	4 (1.0)
Diagnostic examination of, eg, esophagus, stomach, using an endoscope	43235	954 (50)	1584 (928-2596)	472 (49.5)	155 (16.2)	21 (2.2)
Biopsy of the esophagus, stomach, and/or upper small bowel using an endoscope	43239	1015 (50)	1640 (982-2778)	510 (50.2)	144 (14.2)	26 (2.6)
Diagnostic examination of large bowel using an endoscope	45378	971 (50)	1635 (993-2741)	492 (50.7)	155 (16.0)	27 (2.8)
Biopsy of large bowel using an endoscope	45380	935 (50)	2005 (1244-3170)	448 (47.9)	124 (13.3)	20 (2.1)
Removal of polyps or growths of large bowel using an endoscope	45385	898 (50)	1984 (1243-3416)	439 (48.9)	115 (12.8)	21 (2.3)
Ultrasonographic examination of lower large bowel using an endoscope	45391	268 (46)	1492 (1148-2329)	145 (54.1)	63 (23.5)	10 (3.7)
Removal of gallbladder using an endoscope	47562	677 (49)	6923 (4496-13482)	319 (47.1)	109 (16.1)	5 (0.7)
Repair of groin hernia, patient aged ≥5 y	49505	666 (49)	5163 (3058-9644)	310 (46.5)	105 (15.8)	4 (0.6)
Biopsy of prostate gland	55700	777 (49)	1794 (916-3280)	402 (51.7)	149 (19.2)	10 (1.3)
Surgical removal of prostate and surrounding lymph nodes using an endoscope	55866	188 (46)	11 790 (7761-21 041)	95 (50.5)	36 (19.1)	2 (1.1)
Routine obstetric care for vaginal delivery, including predelivery and postdelivery care	59400	127 (36)	3322 (2400-4514)	79 (62.2)	28 (22.0)	3 (2.4)
Routine obstetric care for cesarean delivery, including predelivery and postdelivery care	59510	108 (32)	3650 (2665-5571)	74 (68.5)	27 (25.0)	4 (3.7)
Routine obstetric care for vaginal delivery after prior cesarean delivery	59610	51 (28)	4010 (2624-6157)	29 (56.9)	10 (19.6)	2 (3.9)
Injection of substance into spinal canal of lower back using imaging guidance	62322	609 (48)	900 (639-1453)	369 (60.6)	134 (22.0)	23 (3.8)
Injection of substance into spinal canal of lower back using imaging guidance	62323	968 (50)	1177 (746-1930)	561 (58.0)	158 (16.3)	30 (3.1)
Injections of drug into lower or sacral spine nerve root using imaging guidance	64483	901 (50)	1244 (804-2005)	511 (56.7)	186 (20.6)	24 (2.7)
Removal of recurring cataract in lens capsule using laser	66821	329 (46)	1048 (608-1565)	173 (52.6)	72 (21.9)	12 (3.6)
Removal of cataract with insertion of lens	66984	448 (49)	3678 (2344-6159)	213 (47.5)	47 (10.5)	9 (2.0)
Electrocardiogram, routine, with interpretation and report	93000	398 (48)	125 (52-144)	182 (45.7)	61 (15.3)	3 (0.8)
Insertion of catheter into left side of heart for diagnosis	93452	537 (46)	5977 (3816-8617)	329 (61.3)	77 (14.3)	20 (3.7)
Sleep study	95810	1087 (50)	2476 (1596-3534.7)	529 (48.7)	111 (10.2)	25 (2.3)
Physical therapy, therapeutic exercise	97110	1426 (51)	87.4 (63-129.8)	716 (50.2)	161 (11.3)	53 (3.7)

Abbreviations: *CPT*, *Current Procedural Terminology*; CT, computed tomography; DRG, diagnosis related group; MCC, Merkel cell carcinoma; MRI, magnetic resonance imaging; PSA, prostate-specific antigen; TSH, thyroid-stimulating hormone.

^a^ The description of some services was simplified. There are 74 services listed because 4 were specified by the Centers for Medicare and Medicaid Services as containing 2 *CPT* codes (81000 or 81001, 81002 or 81003, 84153 or 84154, and 62322 or 62323).

^b^ Values indicate the number of hospitals that disclosed negotiated commercial price information for the service and the number of states where these hospitals are located.

^c^ Each hospital has only 1 cash price. The median cash price is the median value among all disclosing hospital cash prices.

^d^ Hospitals usually have multiple commercial negotiated prices because they contract with insurers providing different insurance plans. For each hospital, its median commercial price across all insurance plans was compared with its cash price. The proportion of disclosing hospitals for which the cash price was lower than the median cash price is reported for each service.

^e^ For each hospital, all of its commercial prices were compared with its cash price. The proportion of disclosing hospitals for which the cash price was lower than all of the commercial prices is reported for each service.

^f^ For each hospital, its minimum commercial price across all insurance plans was compared with its cash price. The proportion of disclosing hospitals for which the cash price was equal to the minimum cash price is reported for each service.

The proportion of hospitals that set their cash price below their median commercial negotiated price ranged from 589 of 1534 (38.4%) for *CPT* 80076 (liver function blood test panel) to 74 of 108 (68.5%) for *CPT* 59510 (routine obstetric care for cesarean delivery) (mean [SD], 48.7% [6.4%]). The proportion of hospitals that set their cash price below all of their commercial negotiated prices ranged from 103 of 1444 (7.1%) for *CPT* 81001 (manual urinalysis test with examination using a microscope) to 27 of 108 (25.0%) for *CPT* 59510 (routine obstetric care for cesarean delivery) (mean [SD], 13.7% [4.7%]). Across procedures, between 0.6% of hospitals (4 of 49 hospitals for *CPT* 49505) and 4.3% of hospitals (19 of 48 hospitals for *CPT* 90846) set their cash price exactly equal to their lowest commercial negotiated price (mean [SD], 2.7% [0.8%]).

## Discussion

Across the 70 CMS-specified services, only a mean of 922 hospitals in this economic evaluation had disclosed both their cash price and commercial negotiated price as of July 1, 2021. As evidenced by the negative correlation between the median cash price of a service and the number of hospitals disclosing a price for the service, more expensive services were less likely to be disclosed, which might suggest strategic disclosing decisions. Some hospitals set their cash price comparable to or lower than their commercial negotiated price.

To the extent that more hospitals will disclose prices to comply with the Hospital Price Transparency Final Rule, the cross-hospital variation of cash prices will likely increase. Because of its descriptive nature, this study was unable to identify factors or outcomes associated with the cash price variation. The results of this study, limited to 70 CMS-specified services, may not be generalizable to all hospital services.

In summary, cash prices determined unilaterally by hospitals are often lower than commercial prices negotiated between hospitals and insurers. Uninsured and underinsured patients who choose to take the cash price offered by hospitals might benefit financially.
